# A content analysis of medication adherence material in patient educational resources about gout

**DOI:** 10.1093/rap/rkae042

**Published:** 2024-03-19

**Authors:** Yasaman Emad, Christina Derksen, Keith J Petrie, Nicola Dalbeth

**Affiliations:** Department of Psychological Medicine, University of Auckland, Auckland, New Zealand; Wolfson Institute of Population Health, Queen Mary University of London, London, England; Department of Psychological Medicine, University of Auckland, Auckland, New Zealand; Department of Medicine, University of Auckland, Auckland, New Zealand

**Keywords:** gout, medication adherence, online resources, patient engagement

## Abstract

**Objective:**

This study aimed to investigate how medication adherence is addressed in online gout resources in six countries. We investigated how often adherence was referred to, the strategies suggested to improve patient adherence, and the types of nonadherence that were targeted. We also examined the readability of the adherence material.

**Methods:**

A content analysis was conducted on 151 online gout resources from medical and health organisations in six predominantly English-speaking countries. Two reviewers coded the content of the websites into categories (kappa 0.80). The analysis involved coding the resources for reasons for nonadherence, and adherence-promoting strategies. Flesch-Kincaid Reading Ease scores and word count were also computed.

**Results:**

Out of 151 websites examined, 77 websites discussed medication adherence (51%), with intentional nonadherence being more prevalent than unintentional nonadherence. 67 websites targeted different types of nonadherence, including drug-specific concerns (50%), misconceptions of gout curability and the necessity of medication (16%), forgetfulness (16%), and other practical challenges (5%). Strategies to promote adherence were found in one-third of the websites, with medication education being the most prevalent strategy (17%), followed by healthcare provider engagement (13%) and memory aid strategies (6%). On average, about 11% of the words (89.27, SD = 76.35) in the entire document were focused on adherence. Difficult reading comprehension was found in one-fifth of adherence-related websites.

**Conclusion:**

Findings reveal limited medication adherence coverage and narrow strategies in online gout resources. Improved adherence portrayal is needed for effective gout management through comprehensive strategies and clear, understandable information.

Key messages

- This study revealed the limited focus and accessibility of adherence information in online educational resources.
- The findings underscore the importance of developing strategies that target the main motives behind medication nonadherence.

## Introduction

Low adherence to urate-lowering therapy (ULT) represents a major clinical issue in the management of gout. Recent systematic reviews have shown that continuation rates for ULT are low [1] with a steady number of patients discontinuing treatment after their initial prescription and less than half of people with gout still taking their urate-lowering medication regimen at 12 months [2, 3].

Nonadherence can be classified into two categories: unintentional and intentional. Intentional nonadherence describes the process by which patients decide not to take their medication based on specific beliefs and perceptions about their condition or treatment [4]. For instance, some people with gout may choose not to adhere to their medication regimen out of concern about experiencing adverse side effects. On the other hand, unintentional nonadherence is not a deliberate act of omission, but rather an unplanned behaviour, such as forgetting to take the medication, challenges in obtaining medication refills, or encountering logistical barriers like travel or disrupted routines [5].

Intentional nonadherence poses a significant challenge in effectively managing gout, as it hinders the optimal utilization of urate-lowering therapy and undermines its potential benefits. Recent studies have identified four main reasons behind intentional nonadherence to ULT. The first is r*esisting illness*, which is the desire to feel healthy and maintain a sense of normalcy, and not to be reminded of the fact that the patient has gout. Secondly, *testing treatment*, which is the patient testing the limits of treatment by taking the least amount possible to avoid gout attacks, Thirdly, *drug-related concerns*, include worries about the side effects from the medication, concern that the ULT will lose effectiveness over time, and anxiety about developing drug dependency. Fourthly, *medicine sensitivity* refers to the patient feeling highly sensitive to the effects of ULT [6].

Recent studies have emphasized the significance of online health information seeking as a potential factor that can impact adherence to prescribed medications [7]. The way in which people with gout consume online health information can shape their beliefs surrounding their condition and medication use [8], ultimately impacting their behaviour and level of adherence to prescribed medications [9]. Previous studies show that the majority of patients with gout tend to seek online health information about their condition and medications [10, 11]. However, the readability and comprehensibility of online gout resources have remained fairly understudied. Readability, which encompasses factors such as sentence structure and vocabulary, plays an important role in patient understanding and engagement [12]. Poor readability can create barriers to patients’ ability to comprehend and follow the recommended adherence strategies [13].

To date, while limited studies have explored the topic, a comprehensive examination of how adherence is framed and discussed in online gout patient resources remains lacking. Our study aimed to fill this gap by investigating how medication adherence is addressed in online gout resources, including how often it is mentioned, the types of nonadherence that are targeted, the strategies used to promote adherence and the readability of the provided information. The findings may help identify the gaps in online patient education around adherence behaviour and provide a clearer picture of what is needed to help patients adhere to their ULT.

## Materials and methods

### Data sources

The resources were identified using a Google search in an ‘incognito window’ to avoid personalization of search results based on the computer’s browsing history. The keywords ‘gout’, ‘gout arthritis’, ‘gout treatment’, ‘gout medication’, ‘pills for gout’, ‘gout drugs’, ‘allopurinol’, ‘febuxostat’, ‘probenecid’, and ‘benzbromarone’ were used to perform a separate search for each of the Google domains using the Google advanced search tool. The first 50 search outcomes in each country were reviewed to identify resources from medical and health organisations and collated for further analysis.

### Selection of websites

Information from patient resources for gout was analysed from medical and health organisations, including the World Health Organization (WHO), Food and Drug Administration (FDA), health governing agencies [including Ministry of Health and Primary Health Organization (PHOs)], health and disability non-profit non-governmental organisations (NGOs), National Institutes of Health, health organisations funded by or affiliated to health governing agencies, medical and pharmacological associations, hospitals, universities, and academic institutions.

### Inclusion and exclusion criteria

All included resources provided information on gout and adherence to urate-lowering medications, aimed at people with gout and the public, dated back to 2018 onwards, and were accessible online in six predominantly English-speaking countries encompassing Australia, Canada, Ireland, New Zealand, South Africa, the United States and the United Kingdom. These countries were selected based on previous research that assessed texts in educational materials about gout [14].

Resources were excluded from the content analysis if they only consisted of published articles, e-books, book chapters, interviews, or reports, or needed to be downloaded as doc, docx and pdf document. Other types of resources excluded included PowerPoint slides aimed at health professionals and those that included no information about gout or medication adherence for gout (eg. provided insurance advice for patients). Material that required a paid subscription or creating an account or did not come from a medical/health organization was also excluded.

### Content analysis

A sample of websites were initially reviewed by two reviewers (YE and CD), and 8 categories were agreed upon. Both reviewers (YE and CD) then coded the content of all websites into intentional nonadherence categories. Kappa (SE) was calculated to assess the level of agreement between the two reviewers, and the calculated value was 0.80 (0.03), indicating substantial agreement between the reviewers regarding their assessments of the materials based on the established codes for promoting adherence strategies and the targeted reasons for non-adherence. A total of 151 cases were included in the analysis, and the observed agreement between the reviewers in their assessments of the adherence promotion strategies and nonadherence reasons was 0.93. Any discrepancies in coding were resolved through review by a third author (KP). The analysis involved calculating the frequency and percentage of reasons for nonadherence and the strategies employed to promote adherence within the categories. To facilitate the organization and interpretation of the data, relevant nonadherence categories were further divided into subcategories.

Website material was initially categorised by two reviewers into intentional and unintentional adherence. Unintentional adherence material addressed reasons, such as forgetfulness or physical inaccessibility, while intentional material focused on nonadherence reasons identified in previous studies, such as drug-specific concerns or medication-taking causing disruption of normal life. The reviewers then coded the content of all websites into nine nonadherence categories, including: Perceived disruption of normal life (e.g. if you do not keep your serum urate under control, you may not be able to do something as simple as accompany your kids to the school bus stop or walk your dog), Inconvenience with medication administration (e.g. not easy to take med all the time), Misconception of gout curability and medication necessity (e.g. gout cannot be cured, you need to take your medication lifelong; you need to take your medication everyday), Perceived lack of need when feeling well or without active symptoms/attacks (e.g. you need to keep taking allopurinol even when you have no active symptoms or no gout attacks), Delayed efficacy awareness (e.g. it will take a while to see the benefits of your medication; Allopurinol takes 2 to 3 months to become fully effective), Flare-driven nonadherence (e.g. experiencing flare-ups after initiating allopurinol), Side effects, Nonadherence due to concern about being on the wrong dose and Concerns about medication dependency.

A Flesch-Kincaid Reading Ease score was calculated for the adherence-related text using a web-based readability measurement tool at https://readability-score.com. This score indicates what level of education is typically needed to comprehend a piece of writing. In addition, we quantified the word count of the adherence-related content, using Notepad.

### Ethical statement

Ethical approval was not required for this study, as it involved the analysis of publicly available data, in accordance with the policy of the University of Auckland Human Participants Ethics Committee. The content used in this research was obtained from the public domain, and therefore, individual consent or consent from websites was not obtained.

## Results

The analysis of online gout resources involved an initial search that yielded a total of 270 websites. After excluding irrelevant or duplicate websites, 151 websites met the inclusion criteria and were included in the analysis.

### Frequency and types of nonadherence

Our first objective was to explore how often medication adherence was discussed in online gout resources. Among the 151 websites, we found 77 websites mentioned adherence to urate-lowering medication (50.9%). 67 websites (44.3%) specifically targeted different types of nonadherence and examined the potential reasons associated with this behaviour.

Out of the 151 websites, intentional nonadherence was reported in 66 sites (43.7%), while unintentional nonadherence was mentioned in 30 sites (19.8%).

### Targeted reasons for nonadherence

Next, we conducted an analysis of 67 websites focusing on adherence to urate-lowering medications to explore the specific reasons targeted when discussing nonadherence. The identified reasons were then categorised into four main categories. Firstly, drug-specific concerns emerged as the primary contributing factor, encompassing half of the identified reasons. This category included nonadherence due to experiencing flare-ups after initiating treatment, medication delayed effectiveness, side effects, concerns about being on the wrong dose and anxiety about developing drug dependency. Secondly, misconceptions of gout curability and medication necessity were identified in 24 websites (15.8%). This category encompassed perceived lack of need when feeling well or without active symptoms/attacks and misconception of gout curability. Thirdly, forgetfulness was targeted as a reason for nonadherence in 24 websites (15.8%). Lastly, other factors were targeted in 7 websites that accounted for 4.6% of the identified reasons, including difficulties accessing medication, perceived disruption of normal life, and inconvenience with medication administration. All of the figures are out of 151 (Table 1).

**Table 1.** Proportion of targeted reasons for nonadherence used among websites included in the study (n = 151)^a^

Targeted reasons for nonadherence	*n (%)*
Drug-specific concerns	76 (50.33)
Flare-driven nonadherence (e.g. you may experience flare-ups after initiating allopurinol)	41 (27.15)
Delayed efficacy awareness (e.g. it will take a while to see the benefits of your medication; Allopurinol takes 2 to 3 months to become fully effective)	19 (12.58)
Side effects	10 (6.62)
Nonadherence due to concern about being on the wrong dose	5 (3.31)
Concerns about medication dependency	1 (0.66)
Misconception of gout curability and medication necessity	24 (15.89)
Perceived lack of need when feeling well or without active symptoms/attacks (e.g. you need to keep taking allopurinol even when you have no active symptoms or no gout attacks)	17 (11.25)
Misconception of gout curability (e.g. gout cannot be cured, you need to take your medication lifelong)	7 (4.63)
Forgetfulness	24 (15.89)
Other	7 (4.63)
Inconvenience with medication administration (e.g. it is not easy to take medicine all the time)	4 (2.64)
Perceived disruption of normal life (e.g. if you do not keep your serum urate under control, you may not be able to do something as simple as accompany your kids to the school bus stop or walk your dog)	2 (1.32)
Difficulty accessing medication	1 (0.66)

a The percentages reflect the proportion of websites targeting each reason individually, but not collectively. Since some websites targeted multiple reasons, the sum of percentages may exceed 100%.

### Adherence-promoting strategies

We also identified and categorised strategies utilized in the online gout resources to facilitate adherence. A comprehensive analysis was conducted on the 45 websites that discussed one or more adherence-promoting strategies. Through this analysis, we identified and categorised the following strategies. Firstly, providing medication education was prominently featured in the analysed websites, accounting for almost two-thirds of the strategies (60% or 27). The most prevalent medication education strategy involved explaining the mechanism of action and highlighting the benefits of the prescribed medication, constituting 53% of the identified approaches (or 24 websites). Strategies emphasizing the consequences of medication nonadherence, such as joint damage and kidney stones, were also featured, accounting for almost 7% of the identified approaches (or 3 websites). Secondly, healthcare provider engagement was emphasized by 42% of the analysed websites (or 19 websites), such as advice to contact healthcare providers for support and guidance on medication adherence. Thirdly, memory aid strategies were found to be employed by approximately one-fifth of the analysed websites (or 9 websites). These strategies included establishing a daily medication routine, providing recommendations for optimal medication administration, enhancing medication accessibility and visibility (4% or 2), utilizing reminders or alarms and using a pill box. Lastly, additional strategies to promote adherence included getting regular blood checks, making a treatment or emergency plan, reading medication label instructions, contacting available patient helplines and following instructions from healthcare professionals. All of the figures are out of 45 (Table 2).

**Table 2.** Proportion of strategies used among websites included in the study (n = 151)^a^

Adherence-promoting strategies	*n (%)*
Providing Medication Education	27 (17.88)
Educational emphasis on medication nonadherence symptom consequences	3 (1.98)
Explaining medication mechanism of action and benefit	24 (15.89)
Healthcare provider engagement	
Contacting the healthcare provider	19 (12.58)
Memory Aid Strategies	9 (5.96)
Using a pill box	1 (0.66)
Reminder/Alarm utilisation	2 (1.32)
Improving medication accessibility	2 (1.32)
Optimal medication administration	4 (2.64)
Establishing a daily medication routine	11 (7.28)
Other	8 (5.29)
Contacting Healthline	1 (0.66)
Following given instructions	1 (0.66)
Reading medication label instructions	2 (1.32)
Making a treatment or emergency plan	2 (1.32)
Regular blood check	4 (2.64)

a The percentages reflect the proportion of websites that employed each strategy individually, but not collectively. Since some websites utilise multiple strategies, the sum of percentages may exceed 100%.

### Ontent readability

**Figure 1.**
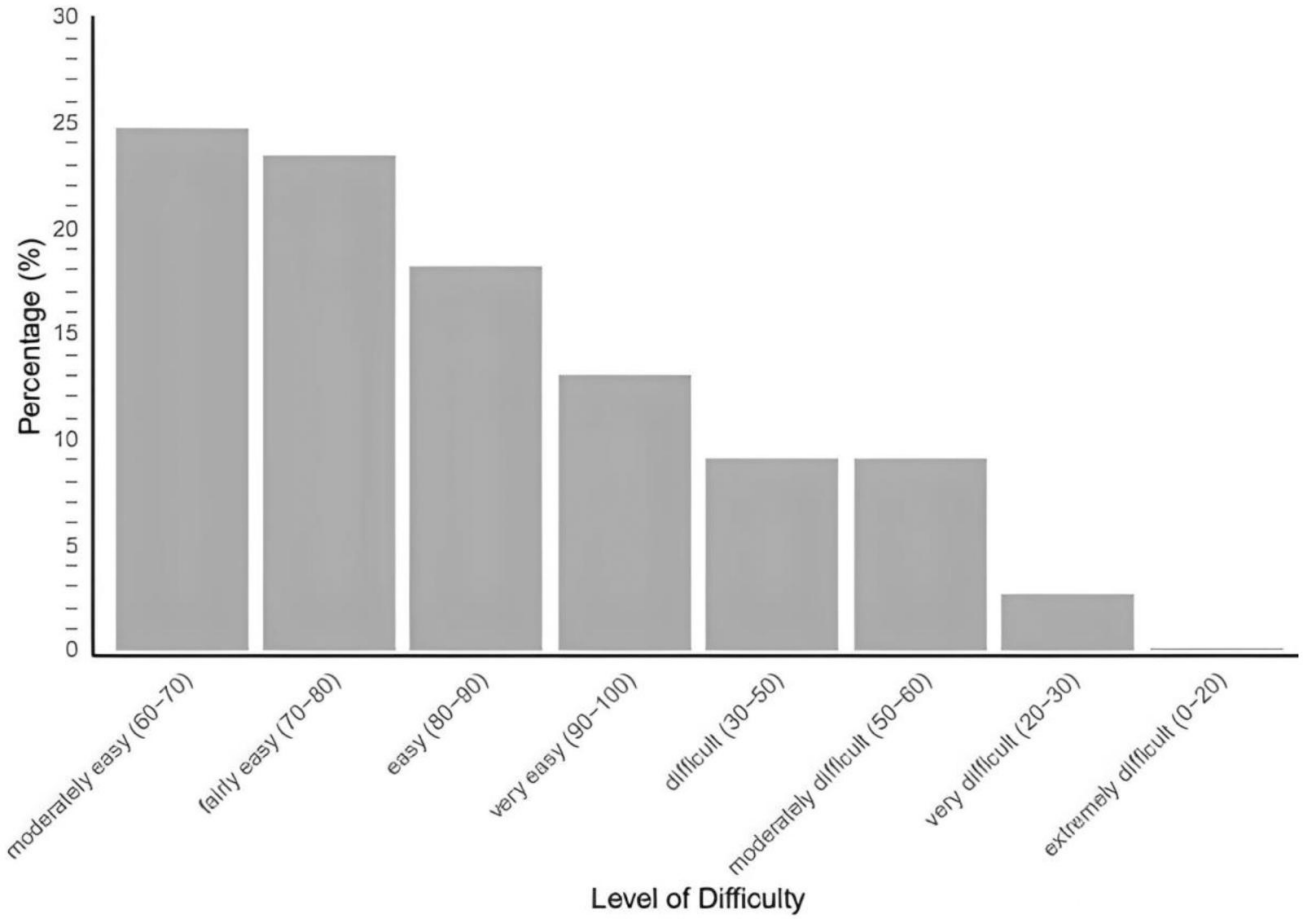
Flesch-Kincaid Reading Ease scoring for websites included in the study (n = 151)

Lastly, we conducted an assessment of content readability and word count for adherence-related information in online gout resources. Our findings revealed wide variations in the readability and comprehensiveness of the content, as evaluated using the Flesch-Kincaid Reading Ease scores. The analysis showed that the majority of content in adherence text fell within the ‘very easy’ to ‘moderately easy’ range (79.2% or 61). However, approximately one-fifth of sections (or 16 websites) were categorised as ‘difficult’, ‘moderately difficult’, and ‘very difficult’ (Fig. 1). This suggests that certain website content may pose challenges for readers, potentially hindering their understanding and engagement with the information. All of the figures are out of 77.

In addition to the Flesch-Kincaid Reading Ease scores, the average word count across the analysed sections was 89.27 (SD = 76.35; Range = 401–11), constituting approximately 11.1% of the mean word count of the evaluated content (X̄ (SD) = 804 (392.6); Range = 2140–156). This indicates that the sections are relatively concise, presenting a moderate amount of information.

## Discussion

To our knowledge, this study represents the first comprehensive analysis of online gout resources with a specific focus on nonadherence to urate-lowering medications. Contrary to the existing literature emphasizing the pivotal role of adherence in optimizing gout management [15], our study found that only half of the websites mentioned medication adherence, highlighting a significant gap in online resources for patients. Even when mentioned, only limited attention was given to adherence as a proportion of word count in this study indicated. Our analysis further revealed that online gout resources predominantly targeted intentional nonadherence, with less attention given to unintentional barriers. This focus signifies a growing recognition of intentional nonadherence as a major cause of non-adherent behaviour in gout, as it is in other chronic illnesses [6].

In this study, we examined the focus of online material about gout nonadherence. Aligning with research about the causes of non-adherence [16, 17], drug-specific concerns emerged as a prominent category of the material for patients, encompassing a significant proportion of identified factors. Within this category, experiencing flare-ups after initiating medication and perceiving delayed efficacy were commonly discussed, recognizing that patients may modify or discontinue their medication during flares, possibly perceiving it as ineffective [18, 19].

The necessity of ongoing medication even in the absence of symptoms was another important focus in websites discussing nonadherence. These findings are in line with previous studies, emphasizing the importance of patient education on the chronic nature of gout and the importance of consistent adherence to prevent future attacks and long-term complications [20]. By fostering a comprehensive understanding of gout as a chronic condition and elucidating the benefits of sustained medication use, websites can foster informed decision-making among patients and promote a long-term commitment to therapy [21, 22].

The analysis of websites also highlighted important gaps in online patient resources on adherence to urate-lowering medication. Previous work has identified that many patients do not adhere to urate-lowering medication out of a desire to feel healthy and maintain a sense of normalcy [6]. Framing ULT as a way of maintaining normal functioning and activity without the interruption of gout attacks may be an important way to address these patient concerns. Another important reason for patient nonadherence is a deliberate strategy to test their treatment and see if they can get away with less or no treatment. This aspect is rarely addressed in the current material on nonadherence to gout medications and could be a focus of increased attention.

Unintentional factors could also receive more consideration in the website material. Studies suggest forgetfulness is a challenge faced by many people with gout in adhering to medication regimens [9, 11]. However, our analysis reveals that memory aid strategies were only discussed in roughly one-fifth of websites despite their demonstrated effectiveness in promoting medication adherence [23]. Other strategies such as the use of visible location of the medication, coordinating medicine-taking with the patient’s daily routine and the development of ‘if-then’ plans and utilizing social support could be usefully incorporated into the website materials to address common unintentional causes of nonadherence [24, 25, 26].

This study also aimed at examining the content readability of online gout resources to provide primary insights into the accessibility and comprehensiveness of the information presented. Our data revealed a range of readability levels, with about a fifth of websites containing material that was classified as ‘difficult’ or ‘very difficult’. This indicates that some parts of the online resources may pose comprehension challenges for readers, hindering their engagement and understanding of the information [13].

Limitations of the research should be acknowledged. Firstly, our analysis was restricted to predominantly English-speaking countries and so may not be generalisable to patient websites presented in other languages. Secondly, there are currently no guidelines for what types of material on nonadherence should be covered on patient websites, which perhaps explains some of the variability in material. Given the large number of patients that access material about their illness and medications online, this should be the focus of future work. Thirdly while the study looked at how easy the website material was to read; readability scores alone cannot serve as an indicator of the quality or comprehensiveness of the information provided. Our analysis primarily focused on the type of information available on the websites, without assessing the accuracy or scientific rigor of the content. Finally, this study focused on patient-facing educational resources and did not include an analysis of resources for healthcare professionals such as management guidelines or reference texts. Future work would include a similar analysis of gout management guidelines and specifically evaluating any recommendations on adherence optimisation.

In conclusion, this study presented a comprehensive content analysis of online gout resources, with a specific emphasis on intentional nonadherence to urate-lowering medications. The study showed that around half of websites providing patient information on gout did not cover adherence or provide any strategies to help patients keep to their medication regimen. The findings also identified the need to include more content that addresses common patients' beliefs and perceptions related to their urate-lowering medication, which often drives intentional nonadherence behaviour. Websites could also be improved with greater attention to unintentional factors such as forgetting and through improving readability to help patients with lower health literacy.

## Data Availability

The data underlying this article will be shared on reasonable request to the corresponding author.
